# Tuberculosis in prisons: an integrated prevention and care cascade for a persistent structural epidemic

**DOI:** 10.3389/fpubh.2026.1892055

**Published:** 2026-07-08

**Authors:** Jaen Cagua-Ordoñez, Juan Marcos Parise-Vasco, Jaime Angamarca-Iguago, Natasha Bella Fuentes-Tumbaco, Daniel Simancas-Racines

**Affiliations:** 1 Center for Evidence, Implementation, and Health Decision-Making (CIDES), Facultad de Ciencias de la Salud y Bienestar Humano, Universidad Tecnológica Indoamérica, Ambato, Ecuador; 2 Laboratorio clínico, Unidad de Apoyo Diagnóstico, Hospital Pablo Arturo Suárez, Quito, Ecuador

**Keywords:** active case finding, computer-aided detection, continuity of care, health equity, prisons, tuberculosis

## Abstract

Tuberculosis (TB) remains disproportionately concentrated in prison populations worldwide, driven by overcrowding, poor ventilation, HIV co-infection, delayed diagnosis, treatment interruption, social vulnerability, and weak integration between correctional health services and national TB programs. This structured narrative review synthesizes current evidence across the prison TB prevention and care cascade, including screening, diagnosis, treatment, transmission reduction, TB/HIV integration, surveillance, and continuity after release. We conducted an expert-led narrative synthesis informed by systematic-review principles, distinguishing direct prison-specific evidence from mathematical modeling, policy guidance, systematic reviews, and indirect evidence from adjacent fields such as HIV care and TB infection treatment. Symptom screening alone has limited sensitivity in high-burden prisons. Digital chest radiography with computer-aided detection can improve case detection when linked to confirmatory molecular testing and treatment capacity. Xpert MTB/RIF or Ultra are central to rapid confirmation and rifampicin-resistance detection. Shorter rifamycin-based regimens for TB infection generally achieve higher completion than longer isoniazid-based regimens, while shorter all-oral MDR/RR-TB regimens may offer programmatic advantages but remain insufficiently studied in correctional settings. Ventilation improvement, safer spaces, peer-supported case detection, and integrated TB/HIV services are important components of transmission reduction. Decongestion may have substantial population-level impact, but this conclusion is supported mainly by mathematical modeling and should be interpreted as a projected effect rather than direct prison-specific causal evidence. Post-release continuity remains a major implementation gap, with limited direct evidence for active TB interventions. Sustainable prison TB control requires moving from isolated activities to an integrated, measurable prison–community cascade linking risk-adapted screening, rapid diagnosis, effective treatment, environmental controls, data systems, and confirmed linkage to community care after release.

## 1 Introduction

Tuberculosis (TB) remains disproportionately concentrated in prisons worldwide. A global systematic review and meta-analysis by Cords and colleagues found a high burden of TB disease and *M. tuberculosis* infection among incarcerated populations, with substantial heterogeneity across regions, income levels, background TB burden, and prison system characteristics (1). This excess burden is large in both relative and absolute terms. The same review estimated a pooled incidence of *M. tuberculosis* infection of approximately 15.0 new infections per 100 person-years among incarcerated populations, indicating intense ongoing transmission in many correctional settings (1). TB disease incidence also varies markedly across regions and prison systems, while earlier systematic review evidence by Baussano and colleagues estimated that TB incidence in prisons was, on average, approximately 23-fold higher than in corresponding general populations (2). This excess burden is not explained by individual risk alone; it reflects the convergence of crowding, poor ventilation, malnutrition, HIV co-infection, delayed diagnosis, disrupted treatment, population turnover, and weak integration between correctional health services and national TB programs. In epidemiological terms, prisons can function as TB amplifiers, concentrating exposure and transmission within facilities and contributing to transmission beyond prison walls through releases, transfers, visitors, and occupational exposure among staff (3, 4).

The scale and turnover of the global prison population make prison TB a public health issue of broad relevance. More than 10 million people are incarcerated globally at any given time, with recent estimates exceeding 11 million, and many more pass through correctional systems each year because of short sentences, transfers, and pre-trial detention (5). Reviews and comparative descriptions from countries with large prison populations—including Brazil, the United States, China, Russia, and India—have identified insufficient prison–community coordination, weak notification systems, and fragmented continuity of care as recurring weaknesses across settings (6). Historical experience from post-Soviet Eastern Europe further illustrates the potential for prisons to amplify national TB and multidrug-resistant or rifampicin-resistant TB (MDR/RR-TB) epidemics when prison health systems are under-resourced and poorly integrated with civilian TB programs (7, 8). Ecological analyses linking mass incarceration with increased TB and MDR-TB incidence in Eastern Europe and Central Asia reinforce a central epidemiological principle: prisons are not closed systems, but dynamic interfaces between incarcerated populations, staff, visitors, health services, and surrounding communities (9, 10).

Prison populations have long been recognized by scientific and policy communities as a key vulnerable group requiring prioritized TB prevention and care. Guidance from the CDC for correctional and detention facilities, ECDC technical guidance, WHO recommendations, Stop TB Partnership frameworks, and major systematic reviews have consistently emphasized entry and periodic screening, active case finding, prompt diagnostic confirmation, treatment support, infection prevention and control, discharge or release planning, and coordination between prison and community health services (11). Yet implementation remains inconsistent, particularly where overcrowding, fragmented governance, limited laboratory access, incomplete data systems, weak notification mechanisms, and poor post-release linkage persist. Thus, the implementation gap is not simply the absence of recommendations, but the failure to operationalize them as an integrated, measurable, and accountable prison–community cascade (7, 12).

Recent advances have expanded the technical options available for prison TB control across the prevention and care cascade. These include risk-adapted entry and periodic screening, digital chest radiography with computer-aided detection, rapid molecular confirmation with Xpert MTB/RIF or Ultra, shorter regimens for TB infection, all-oral MDR/RR-TB treatment, integrated TB/HIV services including antiretroviral therapy and TB preventive treatment, peer-supported case detection, environmental interventions, and structured post-release continuity mechanisms. However, the directness of evidence varies across interventions: CXR/CAD, peer-supported case detection, and some TB infection treatment studies have been evaluated in correctional settings, whereas newer MDR/RR-TB regimens, LF-LAM pathways, and some TB/HIV integration strategies rely more heavily on programmatic guidance or indirect evidence (13–16). Population-level effects of decongestion and some environmental interventions should also be interpreted carefully, because much of the supporting evidence comes from mathematical modeling and mechanistic reasoning rather than direct prison-based intervention trials. Tools do not control TB unless they are embedded in systems that ensure screening completion, diagnostic confirmation, treatment initiation, infection prevention action, transfer communication, release planning, community linkage, and outcome monitoring. The unresolved challenge is how to combine these tools into context-adapted systems that remain functional across incarceration, transfer, release, and reintegration into community care (17, 18).

This structured narrative review synthesizes current evidence across the prison TB prevention and care cascade, from structural determinants and screening to diagnostic confirmation, treatment, prevention, environmental control, surveillance, and post-release continuity. Evidence is interpreted with attention to study design, geographic context, and directness to prison settings, distinguishing direct prison-specific empirical studies from mathematical modeling, systematic and narrative reviews, policy guidance, and indirect evidence from adjacent fields. We argue that persistent failure in prison TB control is driven not only by delayed case detection, but by the interaction of structural prison conditions, incomplete diagnostic pathways, treatment interruption, social vulnerability, weak prison-health system integration, and poor continuity of care after release. Sustainable control therefore requires moving from isolated biomedical interventions toward an integrated prison–community cascade that is measurable, context-adapted, and accountable.

## 2 Methods

This article was designed as an expert-led structured narrative review informed by systematic-review principles. The objective was to synthesize and interpret current evidence across the prison TB prevention and care pathway, with emphasis on clinical, epidemiological, implementation, and policy relevance. The review was structured to provide an evidence-informed framework for prison TB control rather than pooled estimates of intervention effectiveness.

A predefined core corpus of peer-reviewed and policy literature on TB in prisons was used as the foundation for the review. This corpus included original epidemiological studies, screening and diagnostic studies, treatment and continuity-of-care studies, mathematical modeling analyses, economic evaluations, systematic and narrative reviews, and policy or technical guidance. Sources were identified from PubMed/MEDLINE, Scopus, Web of Science, Google Scholar, reference lists of key reviews and original studies, and technical guidance documents from relevant public health organizations. Additional policy and technical documents were retrieved from the websites or repositories of the World Health Organization, the Centers for Disease Control and Prevention, the European Centre for Disease Prevention and Control, and the Stop TB Partnership. Searches combined terms related to tuberculosis and correctional settings, including “tuberculosis,” “TB,” “prison,” “jail,” “detention,” “correctional facility,” “incarcerated,” “active case finding,” “screening,” “Xpert,” “Xpert MTB/RIF,” “Xpert Ultra,” “computer-aided detection,” “digital chest radiography,” “TB preventive treatment,” “latent tuberculosis infection,” “MDR-TB,” “MDR/RR-TB,” “HIV,” “LF-LAM,” “ventilation,” “overcrowding,” “decongestion,” “continuity of care,” and “post-release continuity of care.” Evidence was considered through May 2026.

Eligible evidence included studies and guidance addressing TB disease, TB infection, screening, diagnosis, treatment, prevention, transmission reduction, surveillance, or continuity of care in prisons, jails, detention centers, or correctional facilities. Evidence from non-prison settings was considered only when it addressed mechanisms or implementation challenges directly relevant to prison TB control, such as rapid molecular diagnosis, LF-LAM testing among people living with HIV, TB infection treatment, airborne infection prevention, or continuity of care after institutional release. Studies or reports without a clear link to TB control, prison health, congregate settings, or prison–community care transitions were not used to support core conclusions.

Additional sources were identified through structured gap analysis, reference checking, and targeted searches focused on themes insufficiently covered by the core corpus. These themes included post-release continuity of care, MDR/RR-TB, LF-LAM, TB/HIV integration, ventilation, decongestion, TB infection treatment, cost-effectiveness, staff occupational risk, data systems, and implementation science. Evidence from adjacent fields, including HIV care and substance use treatment continuity, was included only when it addressed mechanisms or implementation barriers relevant to prison–community transitions; such evidence was interpreted as indirect and was not used to estimate prison-specific TB intervention effects.

Each source was classified by study design, geographic setting, prison TB domain, population, intervention or exposure, outcome focus, and evidence type. Evidence was interpreted narratively according to its directness, design, setting, and relevance to prison TB control. Direct prison-specific empirical studies were distinguished from mathematical modeling, economic evaluations, diagnostic accuracy studies, systematic reviews, narrative reviews, policy guidance, and indirect evidence extrapolated from non-prison populations or adjacent health conditions. These evidence types were not treated as equivalent; each was used according to the type of inference it could reasonably support. This classification was used to avoid treating direct prison-based empirical studies, model-based projections, policy recommendations, and indirect evidence from adjacent fields as equivalent forms of evidence.

Because this was a structured narrative review rather than a systematic review or scoping review, the protocol was not registered in PROSPERO and we did not perform duplicate independent screening, formal risk-of-bias assessment, quantitative meta-analysis, GRADE certainty assessment, or produce a PRISMA flow diagram. The search was not designed to be exhaustive in the manner of a systematic review, and no fully reproducible search log was generated. Findings are therefore presented as thematic synthesis rather than pooled effect estimates. Where conclusions relied on modeling, indirect evidence, partially prison-specific evidence, or expert interpretation, this is stated explicitly in the text.

The synthesis was organized around the prison TB prevention and care cascade: structural determinants of transmission, screening, diagnostic confirmation, treatment, prevention and transmission reduction, post-release continuity, implementation gaps, integrated program strategies, and policy implications.

## 3 Prisons as structural amplifiers of tuberculosis

The high burden of TB in prisons is well documented and justifies prioritization within national TB control strategies. However, prison TB is not only a problem of delayed diagnosis or inadequate treatment. It is also shaped by the physical environment of detention, the organization of prison health services, the social vulnerability of incarcerated populations, and the weak integration between correctional and community health systems (18, 19). Clinical interventions are necessary, but they are unlikely to achieve sustained transmission control unless implemented alongside measures that reduce exposure intensity, improve air quality, ensure continuity of care, and address the institutional conditions in which transmission occurs (20, 21).

### 3.1 Overcrowding as the primary physical determinant

TB is transmitted through the air, and transmission risk increases with the concentration of infectious aerosols in shared spaces and the duration of exposure. Many prisons combine high occupancy density, prolonged indoor exposure, inadequate ventilation, and delays in separating or treating infectious individuals. These conditions increase the probability that a person with undiagnosed pulmonary TB will transmit infection to others before detection (22).

Mathematical modeling studies provide consistent support for overcrowding as a major determinant of prison TB transmission. Legrand and colleagues modeled intervention scenarios in heavily overcrowded Brazilian prisons and found that, in these models, decongestion—reducing prison population density—had the greatest projected impact among the interventions assessed, exceeding the projected effect of improved case detection, treatment, or ventilation when implemented in isolation (19). Urrego and colleagues similarly found that neither earlier diagnosis nor improved ventilation alone was sufficient to reduce transmission below epidemic thresholds in severely overcrowded settings; combinations involving ventilation, early diagnosis, and/or decongestion were required to achieve meaningful projected control (18). Modeling based on Malaysian prison data reached broadly consistent conclusions (23).

These studies should be interpreted as modeling evidence rather than direct empirical proof of intervention effectiveness. Nonetheless, their consistency across settings supports a central programmatic implication: overcrowding is not merely a contextual barrier to TB control, but a transmission driver that can limit the effectiveness of even well-designed clinical interventions. In this sense, decongestion should be understood not only as an administrative or legal issue, but as a potential airborne infection prevention intervention supported primarily by mathematical modeling, mechanistic plausibility, and consistency across modeled settings. Its expected impact should therefore be interpreted as a projected population-level effect, and decongestion should be implemented as part of a broader package that includes early diagnosis, effective treatment, ventilation improvement, separation of infectious cases when feasible, and continuity of care.

### 3.2 The prison–community interface as an epidemiological bridge

Prisons are dynamically connected to surrounding communities through admission, release, transfer, staff movement, visitors, and health-service referrals. This mobility creates a bidirectional epidemiological bridge: people with TB may enter prisons from the community, transmission may be amplified inside facilities, and individuals with undiagnosed or incompletely treated TB may return to the community after release (7, 21, 24).

Ecological analyses from Eastern Europe and Central Asia suggest that large-scale incarceration and weak prison TB control may influence national TB and MDR-TB dynamics (6). Comparative work across countries with large prison populations has similarly identified insufficient prison–community coordination as a recurring weakness, including gaps in notification systems, referral mechanisms, and recognition of incarceration as a relevant exposure history (25). These findings indicate that prison TB control should not be treated as a closed institutional problem, but as a component of national TB prevention and care. Consequently, prison TB indicators should be integrated into national TB surveillance rather than reported as a parallel or isolated correctional health problem (26).

### 3.3 Social vulnerability as a compounding determinant

Incarcerated populations are disproportionately affected by social and structural determinants associated with TB risk, including poverty, unstable housing, substance use disorders, migration, limited prior access to health care, and, in some countries, racial or ethnic marginalization. These factors increase the likelihood of TB exposure before incarceration and may also reduce the probability of treatment completion after transfer or release (26–28).

Recent reviews have identified these social determinants as recurring contributors to prison TB burden, alongside operational barriers within prison health systems (10). Their interaction with overcrowding, delayed diagnosis, limited infection prevention infrastructure, and poor post-release linkage creates a risk profile that cannot be fully addressed by clinical services alone (20). These determinants affect not only baseline TB risk, but also every step of the care cascade: symptom reporting, access to testing, treatment adherence, release planning, and linkage to community services (29). Effective TB control in prisons therefore requires coordinated action across health, correctional, social welfare, housing, and community-based services (30).

### 3.4 HIV co-infection as a biological amplifier

In prison systems with high HIV prevalence, particularly in parts of sub-Saharan Africa and other high HIV/TB burden settings, TB transmission and disease progression are intensified by immunosuppression. People living with HIV have a higher risk of progression from TB infection to active disease, and delayed diagnosis in this group can contribute to both individual morbidity and ongoing institutional transmission (31).

Transmission modeling from South African correctional settings suggests that HIV-positive incarcerated persons may contribute disproportionately to TB incidence within prisons because of increased susceptibility and faster progression to disease (32, 33). This creates a reinforcing cycle: HIV increases individual risk of TB disease; higher TB incidence increases exposure risk within the prison; and ongoing exposure further increases TB incidence among both HIV-positive and HIV-negative incarcerated persons. From a health-systems perspective, this supports integrated prison TB/HIV services that combine HIV testing, antiretroviral therapy, TB screening and prevention, and coordinated follow-up after transfer or release (34, 35).

### 3.5 Molecular evidence of within-prison transmission

Genomic and molecular epidemiology studies have strengthened the evidence that prisons are not only receiving TB cases from the community but also sustaining transmission internally. Whole-genome sequencing studies in southern Brazil identified clustering of *M. tuberculosis* isolates among incarcerated persons, consistent with recent transmission within correctional settings (36, 37). Importantly, clustering included both drug-susceptible and drug-resistant strains, suggesting that prison environments can facilitate transmission of resistant TB strains as well as drug-susceptible disease (24, 38).

Earlier molecular epidemiological investigations in United States prisons also documented previously unrecognized transmission chains, indicating that individuals may acquire or incubate TB within correctional facilities before clinical detection (39, 40). Taken together, these studies support the conclusion that within-prison transmission has been empirically documented across different settings, rather than being only a theoretical concern.

## 4 Screening strategies: matching tools to burden, risk, and capacity

TB screening in prisons has both clinical and public health objectives. Clinically, it aims to identify people with active TB and link them promptly to diagnostic confirmation and treatment. Epidemiologically, it seeks to reduce the pool of undetected infectious disease and thereby interrupt transmission within high-risk congregate settings. The effectiveness of any screening strategy depends not only on sensitivity and specificity, but also on operational feasibility, confirmatory testing capacity, treatment initiation, infection prevention, and the epidemiological context in which screening is implemented (41, 42).

Symptom screening remains attractive because it is simple, inexpensive, and feasible in most correctional systems. However, symptom screening alone has limited sensitivity for active TB in high-burden prisons, where asymptomatic or minimally symptomatic disease may contribute to ongoing transmission. Reliance on cough, fever, night sweats, or weight loss as the primary screening tool can therefore miss infectious cases, particularly when symptom reporting is affected by stigma, low health literacy, mistrust, or fear of isolation (43, 44).

Digital chest radiography, with or without computer-aided detection, can improve screening yield in high-burden prison settings. Studies from South Africa and other correctional environments suggest that CXR/CAD detects cases that symptom screening alone may miss (13, 45). The operational value of CXR/CAD is greatest when it is embedded in a complete pathway that includes sputum collection, molecular confirmation, treatment initiation, and outcome monitoring. CXR/CAD should be understood as a triage strategy, not a diagnostic endpoint (46).

Periodic active case finding may be necessary in prisons with ongoing transmission. Entry screening alone cannot address cases that develop after admission, are missed at baseline, or arise during prolonged incarceration. Serial mass screening studies from high-burden settings, including Brazil and Bangladesh, indicate that repeated screening can identify additional cases and may reduce facility-level prevalence when linked to treatment (47, 48). The optimal interval for repeat screening is not universal and should depend on TB burden, facility turnover, occupancy density, recent outbreaks, HIV prevalence, and available resources (49) (Table 1).

**Table 1:** Evidence map for prison TB control interventions.

Domain	Intervention or exposure	Key references	Main evidence type	Directness to prisons	Main finding	Evidence caveat	Programmatic implication
Epidemiological burden	Prison incarceration as TB amplifier	Cords et al. (1); Vinkeles Melchers et al. (103); Haeusler et al. (12); Busatto et al. (6); Coninx et al. (104)	Meta-analysis, systematic reviews, narrative reviews	Direct	TB incidence is substantially higher in incarcerated populations than in general populations; prisons can amplify community transmission	Estimates vary by region, background burden, prison type, and surveillance quality	Include prisons explicitly in national TB strategic plans and surveillance
Structural determinants	Overcrowding/decongestion	Legrand et al. (19); Urrego et al. (18); Naning et al. (23); Mahawan et al. (89); Stuckler et al. (9)	Mathematical modeling, ecological analysis	Moderate	Reducing occupancy is projected to substantially reduce transmission, often more than isolated clinical interventions	Modeling assumptions vary; direct intervention studies are scarce	Monitor prison density as an infection-prevention indicator
Screening	Symptom screening	Kim et al. (45); Velen et al. (13); Vinkeles Melchers et al. (103); Haeusler et al. (12)	Empirical studies, systematic reviews	Direct	Symptom screening alone has limited sensitivity in high-burden prisons	Performance depends on symptom reporting, HIV prevalence, disease stage, and screening frequency	Do not use as sole strategy in high-transmission settings
Screening	CXR/CAD	Velen et al. (13); Kim et al. (45); Charalambous et al. (20); Chukwuogo et al. (105); Melendez et al. (106)	Diagnostic accuracy, implementation studies, policy review	Direct/moderate	CXR/CAD improves screening yield and throughput when linked to confirmatory testing	CAD thresholds require local calibration; yield does not equal treatment completion	Use as triage tool when Xpert and treatment capacity are available
Screening strategy	Serial/periodic active case finding	de Araujo et al. (47); Banu et al. (48); Haeusler et al. (12); Charalambous et al. (20)	Prospective/serial screening studies, systematic review	Direct	Periodic active case finding detects cases missed by entry screening and may reduce facility burden	Optimal interval is uncertain; impact depends on linkage to treatment and prevention	Use repeated screening in prisons with ongoing transmission
Peer-supported case detection	Inmate peer educators	Adane et al. (17); Haeusler et al. (12); Charalambous et al. (20)	Cluster-randomized trial, reviews	Direct	Peer educators can increase TB case detection in resource-limited prisons	Case detection, not transmission or mortality, was primary endpoint	Use as complement to professional health services, not substitute
Diagnosis	Xpert MTB/RIF/Ultra and NAATs	WHO (56); Donkeng-Donfack et al. (107); Nguyen et al. (108); Pape et al. (109)	Guidelines, diagnostic studies, modeling	Partly direct	Rapid molecular tests shorten time to confirmation and detect rifampicin resistance	Prison-specific algorithm studies are limited	Embed NAATs in screening-to-treatment cascade
Diagnostic efficiency	CXR/CAD-to-Xpert and sputum pooling	Codlin et al. (110); Zeng et al. (67); Sembiring et al. (68); Donkeng-Donfack et al. (107)	Modeling, validation, economic analyses	Indirect/moderate	Pooling and CAD triage can expand molecular testing under constrained budgets	Pooling unsuitable for high-risk symptomatic or severely ill individuals	Consider only where deconvolution and result return are rapid
TB/HIV diagnosis	LF-LAM in PLHIV	WHO (49); Herce et al. (91)	Guidelines, indirect evidence	Limited direct	LF-LAM may support diagnosis in selected PLHIV with advanced disease or severe illness	Robust prison-specific accuracy and implementation data are lacking	Evaluate within integrated prison TB/HIV pathways
Drug-susceptible TB treatment	DOT and structured treatment support	Marco et al. (79); Schwitters et al. (76); Dhuria et al. (77); Rodgerd et al. (78)	Cohorts, audits, program evaluations	Direct	Supervision can support adherence, but transfers and release drive losses	DOT quality varies; coercive DOT may undermine trust	Use patient-centered DOT plus transfer/release planning
TB infection treatment	Short rifamycin-based regimens	Matucci et al. (82); Wheeler et al. (83); Lincoln et al. (84); Bock et al. (111); Kowada et al. (51)	Systematic review, cohorts, economic modeling	Direct/moderate	Shorter regimens generally improve completion compared with 9H	Completion is an implementation outcome; prevention efficacy mostly extrapolated	Prefer short regimens when active TB is excluded and interactions manageable
MDR/RR-TB	Short all-oral regimens/BPaLM	WHO (85); Ndjeka et al. (112); Anselmo et al. (36)	Guidelines, programmatic data, molecular epidemiology	Limited direct	Shorter all-oral regimens may reduce interruption risk	Prison-specific implementation and outcomes remain poorly characterized	Require DST, adverse-event monitoring, uninterrupted supply, and release linkage
Ventilation	Natural/mechanical ventilation and safer spaces	Escombe et al. (87); Urrego et al. (18); Legrand et al. (19); CDC (113)	Environmental study, modeling, guidance	Indirect/moderate	Better ventilation reduces aerosol concentration and modeled transmission risk	Escombe is not prison-specific; modeled risk reduction is not direct incidence evidence	Treat ventilation as core structural IPC, not optional infrastructure
Continuity after release	Case management/warm handover	Hatwiinda et al. (73); Marco et al. (79); Fry et al. (80); White et al. (81); Avery et al. (94); Loeliger et al. (93, 114)	Situation analysis, cohorts, RCT for TB infection, HIV proxies	Direct/indirect	Passive referral is insufficient; active transition support improves linkage in adjacent conditions	Few controlled studies for active TB after release	Confirm linkage, not referral alone
Systems accountability	Data systems and surveillance linkage	Dara et al. (7); CDC (113); Herce et al. (91); Busatto et al. (6)	Policy, implementation reviews	Direct logic	Cascade gaps are often undocumented; prison–community data are weakly integrated	Few studies test data-system interventions	Link prison data with national TB surveillance and report cascade indicators

Peer-supported approaches may complement formal screening. In resource-limited prisons, trained peer educators can support symptom recognition, health education, referral, and reduction of stigma. A cluster-randomized trial in Ethiopia showed that trained incarcerated peer educators increased TB case detection compared with passive case finding (17). Peer programs require appropriate training, supervision, confidentiality protections, and non-coercive participation (31).

**Figure 1:**
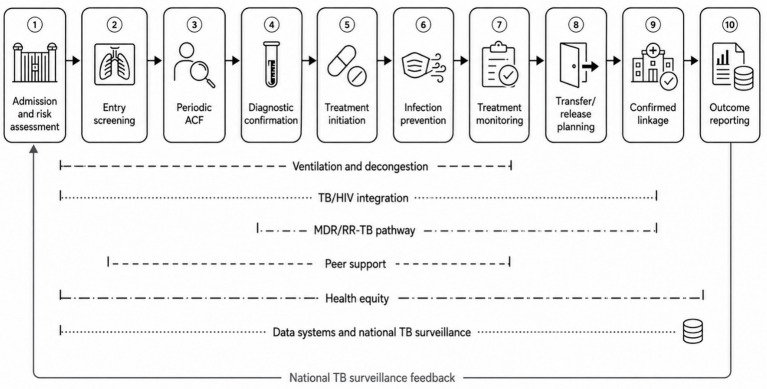
Integrated prison tuberculosis prevention and care cascade. The cascade links admission and risk assessment, entry screening, periodic active case finding (ACF), diagnostic confirmation, treatment initiation, infection prevention, treatment monitoring, transfer/release planning, confirmed linkage to community care, and outcome reporting. Cross-cutting components include ventilation and decongestion, TB/HIV integration, MDR/RR-TB pathways, peer support, health equity, and data systems connected to national TB surveillance. Final outcome reporting should feed back into national TB surveillance and inform subsequent risk assessment and program improvement.

Screening strategies should therefore be risk-stratified. In high-burden, overcrowded prisons, entry screening plus periodic active case finding using CXR/CAD may be appropriate when confirmatory testing and treatment capacity exist. In medium-burden or resource-constrained settings, symptom screening may serve as an initial triage tool, ideally supplemented by targeted CXR or molecular testing for high-risk groups. In low-burden settings, prison-entry testing for TB infection using IGRA or other nationally recommended tests, followed by shorter preventive treatment regimens for eligible individuals, may be more efficient than broad active TB screening (50–52) (Figure 1).

## 5 Diagnostic pathways: from test accuracy to cascade completion

The clinical value of TB screening in prisons depends on timely transition from a positive screen to diagnostic confirmation, treatment initiation, infection prevention action, and public health notification. In many correctional settings, this diagnostic cascade is weakened by delays in specimen collection, inadequate sputum quality, limited laboratory access, transport barriers, delayed result reporting, incomplete documentation, and transfers or releases before treatment is started (53, 54). Each transition creates an opportunity for loss from the care pathway. Diagnostic delay is also epidemiologically consequential: prolonged infectiousness increases the opportunity for transmission, and this effect is amplified in crowded, poorly ventilated congregate settings (7, 55).

Molecular nucleic acid amplification tests have become central to TB diagnosis because they can detect *M. tuberculosis* complex and key drug-resistance markers more rapidly than conventional culture-based workflows. Xpert MTB/RIF detects *M. tuberculosis* DNA and rifampicin resistance in approximately two hours and has replaced or is recommended over smear microscopy as the initial diagnostic test in many high-burden and high-risk settings (56). Xpert MTB/RIF Ultra improves sensitivity, particularly in smear-negative and paucibacillary disease, although this may come with reduced specificity in some clinical contexts, including individuals with previous TB (57, 58).

Evidence directly evaluating Xpert-based diagnostic algorithms in prison populations remains limited. The most relevant trial evidence comes from high-risk congregate or occupational settings with epidemiological similarities to prisons, such as the South African mining industry, where initial Xpert testing reduced time to treatment initiation compared with smear microscopy (59, 60). Although this evidence is indirect, it supports the operational rationale for rapid molecular testing in prison systems, where delayed diagnosis can sustain transmission. In prisons, the programmatic value of NAATs lies not only in faster TB confirmation, but also in earlier detection of rifampicin resistance and faster routing to appropriate drug-susceptibility testing and MDR/RR-TB care (46, 61).

Diagnostic interpretation must account for discordant results. A positive Xpert result with negative culture may reflect low bacillary burden, nonviable organisms after prior TB treatment, culture contamination or loss of viability, or, less commonly, a false-positive molecular result (62, 63). Conversely, a negative Xpert does not fully exclude TB, particularly in people with HIV, paucibacillary disease, poor sputum quality, or extrapulmonary involvement. Diagnostic protocols should specify how to integrate molecular results with symptoms, radiographic findings, HIV status, prior TB history, culture results when available, drug-resistance risk, and clinical judgment (64, 65).

In high-burden prisons using mass or entry screening, testing every screened individual with Xpert may be logistically and financially difficult. A more efficient strategy is to use CXR/CAD as a triage step to identify individuals who require microbiological confirmation (46). People with abnormal CXR findings above a predefined CAD threshold, compatible symptoms, known exposure, HIV infection, prior TB, or other high-risk features should be prioritized for sputum collection and molecular testing. Triage thresholds require local calibration: lower thresholds increase sensitivity but generate more confirmatory testing, while higher thresholds reduce workload but may miss early or paucibacillary disease (66).

Sputum pooling may be considered as an efficiency strategy for high-volume screening campaigns when cartridge capacity is constrained. Evidence from high-burden screening contexts suggests that pooling can reduce cartridge use while maintaining acceptable diagnostic performance when implemented with appropriate pool sizes and follow-up testing of positive pools (67, 68). Pooling should not replace individual molecular testing for people with TB symptoms, abnormal CXR highly suggestive of TB, HIV with advanced immunosuppression, known recent exposure, severe illness, previous TB with concern for recurrence, or high suspicion for drug-resistant TB (69, 70).

Urine lateral flow lipoarabinomannan testing detects mycobacterial antigen and is recommended by WHO for selected people living with HIV, particularly those with advanced immunosuppression, severe illness, or difficulty producing sputum (71). In prisons with high HIV prevalence, LF-LAM can serve as a complementary diagnostic tool for seriously ill or immunocompromised incarcerated persons, especially when sputum-based testing is delayed or not feasible. LF-LAM should not be used as a general screening test for all incarcerated persons. Its value is concentrated in specific clinical subgroups and should be linked to same-day clinical assessment and treatment decisions (72).

The most important diagnostic failures in prisons often occur after a screening or test result is obtained. Hatwiinda and colleagues’ work in Zambian prisons illustrates this problem: people with positive results may be lost before treatment initiation because of incomplete records, weak referral protocols, transfers without documentation, and absence of individual tracking (73). A prison TB diagnostic pathway should therefore define responsibility and documentation at each step: screening result, sputum request, specimen collection, specimen transport, laboratory processing, result communication, clinical review, treatment initiation, drug-susceptibility follow-up, infection prevention action, notification to the national TB program, and outcome reporting (54).

For prisons, diagnostic algorithms should not be evaluated only by test accuracy. They should also be judged by cascade completion: the proportion of people screened, tested, confirmed, started on treatment, linked to ongoing care, and reported to public health authorities. The primary diagnostic outcome should therefore shift from test positivity to cascade completion.

## 6 Treatment in prison settings: from supervised dosing to uninterrupted care

Treatment of TB in prisons occurs within a paradoxical environment. On one hand, incarceration may facilitate structured treatment delivery through predictable routines, regular clinical contact, and supervised medication administration. On the other hand, prison health systems are often affected by overcrowding, staff shortages, medication stock-outs, poor documentation, transfers between facilities, and abrupt release, all of which can interrupt treatment. Effective TB treatment in prisons therefore requires more than access to drugs; it requires a treatment-support system that remains functional across incarceration, transfer, and return to the community (21, 54).

The standard regimen for drug-susceptible pulmonary TB consists of an intensive phase with isoniazid, rifampicin, pyrazinamide, and ethambutol followed by a continuation phase with isoniazid and rifampicin, with duration determined by disease site, drug susceptibility, clinical response, and national guidelines (54). In correctional settings, treatment initiation should be accompanied by standardized documentation of disease site, bacteriological status, drug-susceptibility results, HIV status, weight-based dosing, comorbidities, adverse-event risk, and planned follow-up. Treatment monitoring should include clinical response, bacteriological follow-up when indicated, adverse-event assessment, drug–drug interaction review, and documentation of missed doses or interruptions (21).

Correctional settings can support adherence through directly observed therapy or other structured treatment-support models. However, DOT should not be understood merely as physical observation of pill ingestion. Its effectiveness depends on medication availability, trained staff, adverse-event monitoring, patient education, trust, confidentiality, documentation, and continuity planning. Coercive or poorly implemented DOT may undermine engagement, whereas supportive, patient-centered supervision can improve completion (74, 75).

Observed outcomes vary substantially across prison systems. In Ugandan prisons, Schwitters and colleagues reported reasonable treatment initiation but suboptimal completion, with losses concentrated among people who were transferred or released during treatment (76). An audit of DOTS implementation in Delhi prisons identified medication stock-outs, inadequate staffing, and weak integration with the national TB program as barriers to effective treatment delivery (77). In contrast, evaluations from Thailand found improved outcomes under systematic and supervised prison TB programs, suggesting that implementation quality strongly modifies treatment success (78).

Release from prison is one of the highest-risk transition points in the TB treatment cascade. After release, a person may face unstable housing, poverty, stigma, substance use relapse, competing survival priorities, lack of documentation, difficulty registering at community clinics, and absence of structured adherence support. Evidence from Spain, Russia, Uganda, Zambia, and other settings indicates that the prison–community transition is a recurrent weak point in TB treatment delivery (73, 76, 79, 80).

Release during TB treatment should trigger a predefined continuity protocol rather than an *ad hoc* referral. Minimum elements include documenting the TB diagnosis and regimen, providing a treatment summary to the patient, notifying the receiving TB program before release, scheduling a community appointment, ensuring an initial medication supply when appropriate, assigning a case manager or navigator, and confirming linkage after release. The endpoint should not be referral made, but linkage confirmed (73, 81).

Treatment of TB infection, historically referred to as latent TB infection treatment, is an important prevention strategy in correctional settings when active TB has been excluded and the person has sufficient expected time in custody or a reliable plan for community completion. A systematic review by Matucci and colleagues found three consistent patterns in incarcerated populations: shorter regimens achieved higher completion than nine months of isoniazid; supervised treatment improved completion across regimens; and direct evidence that TB infection treatment prevents future TB specifically in prison populations remains limited, with efficacy largely extrapolated from general-population trials (82).

Shorter rifamycin-based regimens are particularly attractive in prisons because they can often be completed within shorter periods of incarceration. Wheeler and colleagues reported higher completion with three months of weekly rifapentine plus isoniazid than with nine months of isoniazid among California jail inmates, with an acceptable safety profile (83). Earlier jail-based studies also suggested improved completion with shorter regimens, although rifampin-pyrazinamide is no longer favored because of hepatotoxicity concerns (84). In high-transmission prisons, however, TB infection treatment alone is unlikely to control TB unless combined with active case finding, infection prevention, reduced exposure, and continuity after release.

MDR/RR-TB poses a particular threat in prisons because delayed resistance detection, incomplete treatment, overcrowding, and weak continuity systems can sustain transmission and worsen outcomes. Genomic evidence from Brazilian prisons has shown clustering of both drug-susceptible and drug-resistant *M. tuberculosis* strains, supporting the concern that resistant TB may be transmitted within correctional facilities rather than only imported from the community (36).

Current WHO guidance favors shorter, all-oral regimens for eligible patients with MDR/RR-TB, including bedaquiline-based regimens such as BPaLM in appropriate clinical and resistance contexts (85). These regimens are programmatically important for prisons because shorter duration and oral administration may reduce the probability of treatment interruption during incarceration, transfer, or release compared with older 18–24-month injectable-containing regimens. However, prison-specific evidence on newer MDR/RR-TB regimens remains limited. Implementation requires rapid rifampicin resistance detection, access to confirmatory drug-susceptibility testing, assessment for fluoroquinolone resistance, adverse-event monitoring, drug–drug interaction management, uninterrupted drug supply, infection prevention, psychosocial support, and formal linkage to community MDR-TB care before release (86).

## 7 Prevention and transmission reduction: designing multi-component control packages

The prevention of TB transmission in prisons requires more than identifying and treating individual cases. Correctional environments can sustain transmission through high occupancy density, prolonged indoor exposure, poor ventilation, delayed diagnosis, high prevalence of HIV and other comorbidities, and frequent movement between facilities and communities. Biomedical interventions are essential, but their impact is constrained when implemented in environments that continuously generate exposure. Effective prevention requires coordinated action on the physical environment, the diagnostic and treatment cascade, HIV care, TB preventive treatment, and the structural conditions that shape transmission risk (20).

Ventilation reduces the concentration of infectious aerosols in shared indoor air and is therefore a core component of airborne infection prevention. Escombe and colleagues measured air changes per hour in healthcare facilities in Peru and the United Kingdom and found that naturally ventilated spaces with open windows achieved substantially higher ventilation rates than many mechanically ventilated rooms, with modeled reductions in airborne TB transmission risk as ventilation increased (87). Although this study was conducted in healthcare facilities rather than prisons, the underlying principle is relevant to correctional environments: poorly ventilated enclosed spaces increase the probability of airborne transmission.

Translating this principle into prison settings is operationally complex. Security architecture may restrict window design, airflow, and movement. Overcrowding can overwhelm the protective effect of ventilation by increasing the number of infectious and susceptible individuals sharing the same airspace. Existing buildings may be difficult to retrofit, and mechanical systems require maintenance capacity that many prison systems lack. Ventilation reduces exposure risk but does not replace rapid diagnosis, separation of infectious cases, respiratory protection when indicated, or treatment initiation (88).

Overcrowding is one of the most important drivers of TB transmission in prisons. Modeling studies from Brazil, Malaysia, Thailand, and other high-burden settings suggest that reducing prison population density can produce substantial reductions in projected TB transmission, often exceeding the effect of clinical interventions implemented in isolation (18, 23). Decongestion is not a conventional health-sector intervention, but it has direct epidemiological relevance. Potential policy approaches, depending on legal context, include reducing unnecessary pre-trial detention, expanding non-custodial alternatives for low-risk offenses, accelerating legal processes, avoiding incarceration for conditions better managed through health or social services, and ensuring medically appropriate release planning for people at high risk of severe disease (19, 89).

Early detection reduces the duration of untreated infectious pulmonary TB and is therefore a central transmission-prevention strategy. Banu and colleagues provided important prison-based evidence from Dhaka Central Jail, where repeated active case-finding rounds were associated with reductions in facility TB prevalence and estimated transmission, not only increased case detection (48). Conversely, molecular epidemiological work by MacIntyre and colleagues documented previously unrecognized transmission chains in prisons, illustrating how diagnostic delay can allow transmission to continue over time (90). The preventive effect of active case finding depends on the speed with which detected cases move through confirmation, treatment initiation, and infection prevention measures.

In prisons with high HIV prevalence, HIV care is also TB prevention. People living with HIV have increased risk of progression from TB infection to active disease, particularly when HIV is undiagnosed, untreated, or associated with advanced immunosuppression. Integrated TB/HIV services should include routine voluntary HIV testing, rapid ART initiation or continuation, assessment for advanced HIV disease where available, systematic TB evaluation when indicated, TB preventive treatment for eligible individuals after exclusion of active TB, and linkage to community HIV and TB services before release (91).

**Figure 2:**
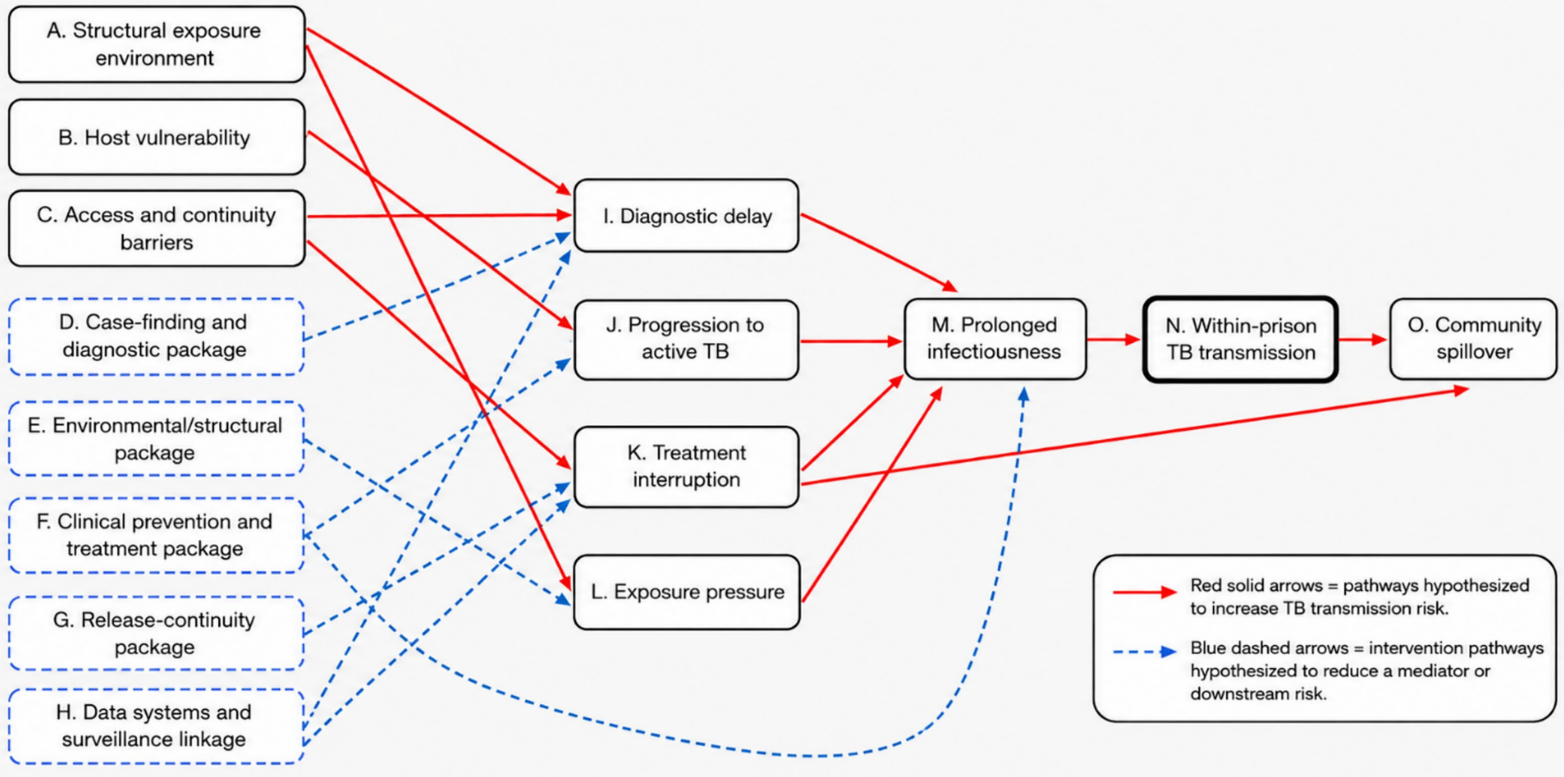
Simplified conceptual directed acyclic graph of mechanisms sustaining tuberculosis transmission in prisons and intervention points for multi-component control. Structural exposure environment includes overcrowding, poor ventilation, prolonged indoor contact, and high-density shared airspace. Host vulnerability includes HIV infection, malnutrition, prior TB, drug-resistance risk, and relevant comorbidities. Access and continuity barriers include high prison turnover, limited laboratory capacity, social vulnerability, incomplete documentation, weak prison–community linkage, and administrative barriers to care. Case-finding and diagnostic package includes active case finding, peer referral, CXR/CAD screening, sputum collection, and Xpert/Ultra confirmation. Environmental/structural package includes ventilation improvement, decongestion, safer spaces, and separation of infectious individuals when feasible. Clinical prevention and treatment package includes ART, TB preventive treatment after active TB has been excluded, effective treatment support, adverse-event monitoring, and uninterrupted drug supply. Release-continuity package includes release planning, warm handover, medication bridging, navigation, transportation support, and confirmed linkage to community TB care. Data systems and surveillance linkage includes prison–community records, standardized transfer forms, national TB notification, treatment outcome reporting, and linkage confirmation.

The strongest argument for multi-component TB prevention in prisons comes from the interaction between interventions. Screening reduces time to detection; treatment reduces infectiousness; ventilation dilutes infectious aerosols; decongestion reduces exposure intensity; ART and TB preventive treatment reduce progression to disease. Their combined effect is more likely to produce sustained control than any isolated intervention. For prison TB programs, prevention packages should be evaluated as system-level interventions, not as collections of disconnected activities (92) (Figure 2).

## 8 Continuity after release: closing the prison–community TB cascade

Continuity of TB care after release is a critical component of prison TB control. Even when diagnosis and treatment are successfully initiated during incarceration, treatment may be interrupted when individuals are transferred between facilities or return to the community without documentation, medication supply, scheduled follow-up, or linkage to a receiving TB program. This transition is clinically important because interruption increases the risk of poor individual outcomes, ongoing infectiousness, recurrence, and development or amplification of drug resistance. It is also epidemiologically important because release reconnects prison-associated TB risk with community transmission networks (21).

Direct evidence on post-release TB care remains limited, but available studies consistently identify the prison–community transition as a vulnerable point in the care pathway. Hatwiinda and colleagues’ situation analysis in Zambian prisons documented failures across multiple steps: transfers between facilities without treatment records, treatment interruption during transfers, absence of discharge planning, and weak coordination with community health systems for released patients (73). Marco and colleagues found that post-release TB treatment adherence in a Spanish DOT cohort was associated with employment status, housing stability, social support, and substance use disorder (79). Qualitative work by Fry and colleagues in Russia described barriers including loss of prison routine, poverty after release, stigma, administrative obstacles to community TB clinic registration, and limited coordination between prison health services and community providers (80). These studies suggest that continuity failure is rarely a single event; it is usually the cumulative result of weak documentation, weak handover, and weak social support.

Because controlled studies of post-release interventions for active TB are scarce, evidence from adjacent conditions can inform program design, while recognizing its indirectness. The HIV literature is relevant because HIV care after release faces similar barriers: medication interruption, loss of structured routines, stigma, competing social needs, and poor linkage to community services. Loeliger and colleagues reported that linkage to HIV care after release was associated with pre-release appointments, case management engagement, and ART coverage during incarceration (93). Avery and colleagues also showed that jail-based case management for people living with HIV improved 12-month retention in care compared with standard referral (94). Evidence from TB infection treatment provides a more TB-specific, though still indirect, basis for continuity interventions. White and colleagues found that nurse case management combined with transportation assistance improved completion of TB infection treatment after release compared with standard referral (81).

A major evidence gap remains: there are no well-powered randomized or rigorously controlled trials evaluating structured post-release case management for active TB among released incarcerated persons, with treatment completion, recurrence, mortality, drug resistance, and post-release transmission as primary outcomes. This limitation should not be interpreted as a reason to defer continuity programs. Rather, programs should be implemented with explicit monitoring and evaluation (Table 2).

**Table 2:** Minimum post-release TB continuity package.

Component	Operational action	Key references	Responsible actor	Measurable indicator	Evidence basis
Pre-release clinical preparation	Update treatment summary, regimen, start date, bacteriology, DST, adverse events, remaining duration	Hatwiinda et al. (73); Fry et al. (80); CDC (113)	Prison health team	% with documented release plan before release	Direct TB implementation evidence and correctional guidance
Warm handover	Direct communication with receiving TB clinic before release, not passive referral	Hatwiinda et al., (73); Marco et al. (79); Fry et al. (80); CDC (113)	Prison TB team + community TB provider	% with receiving provider notified before release	Direct TB evidence of handover failures
Medication bridge	Provide short medication supply when clinically and legally appropriate	Marco et al. (79); CDC (113); Matucci et al. (82)	Prison TB program	% receiving bridge supply when release occurs during treatment	Programmatic logic, adherence evidence
Appointment scheduling	Confirm date, site, contact person, and route to receiving clinic	Hatwiinda et al. (73); White et al. (81); Avery et al. (94)	Prison discharge planner / case manager	% with appointment scheduled before release	Direct TB infection RCT and indirect HIV care evidence
Navigation	Case manager, peer navigator, transportation support, reminders, phone follow-up	White et al. (81); Avery et al. (94); Loeliger et al. (93, 114); Loh et al. (115)	Community TB program or partner organization	% attending first appointment within 7/30 days	TB infection RCT plus HIV/OAT proxy evidence
Social support linkage	Housing, substance use treatment, mental health care, legal/social assistance where available	Fry et al. (80); Marco et al. (79); Loeliger et al. (93); Loh et al. (115)	Community TB/social services	% high-risk patients linked to social support	Social determinants evidence
Feedback and accountability	Receiving provider confirms attendance and accepts responsibility for follow-up	Hatwiinda et al. (73); CDC (113); Herce et al. (91)	Receiving provider / national TB program	% with confirmed linkage, not referral alone	Health-system integration evidence
Enhanced MDR/RR-TB protocol	Medication bridge, DST/adverse-event record, infection prevention instructions, MDR-TB provider handover	WHO (85); Anselmo et al. (36); Ndjeka et al. (112)	Prison MDR-TB team + national DR-TB program	% MDR/RR-TB patients with confirmed MDR-TB care linkage	Limited prison-specific evidence; high-risk clinical logic

## 9 Integrated framework for prison TB control

The evidence synthesized in the preceding sections supports a shift from isolated interventions toward integrated prison TB control strategies. Programs should be designed according to local TB burden, HIV prevalence, prison occupancy, average duration of incarceration, diagnostic capacity, treatment availability, and the strength of prison–community linkage.

No single screening strategy is optimal across all prison systems. In high-burden prisons, especially those with overcrowding or evidence of ongoing transmission, entry screening and periodic active case finding using CXR/CAD should be considered when confirmatory testing and treatment capacity are available. In medium-burden or resource-constrained settings, symptom screening may serve as an initial triage tool, ideally supplemented by targeted CXR or molecular testing for high-risk groups. In low-burden settings, prison-entry testing for TB infection using IGRA or other nationally recommended tests, followed by shorter preventive treatment regimens for eligible individuals, may be more efficient than broad active TB screening (13, 41).

Screening has limited value unless abnormal results lead rapidly to diagnostic confirmation, clinical action, and treatment initiation. Diagnostic algorithms should specify not only which test to use, but who is responsible for acting on the result and within what timeframe. Shorter regimens are especially valuable in correctional settings because incarceration may be brief, transfers are common, and release can interrupt treatment. For TB infection, shorter rifamycin-based regimens such as 3HP or 4R should be prioritized when active TB has been excluded, drug interactions are manageable, and national guidelines support their use (82, 83). For MDR/RR-TB, shorter all-oral regimens may reduce interruption risk but require functioning clinical and public health infrastructure.

TB prevention in prisons requires reducing exposure, not only treating detected cases. Ventilation standards should be incorporated into the design of new correctional facilities and into renovation plans for existing facilities, especially clinics, sputum collection areas, isolation spaces, dormitories, and cells used for people with suspected or confirmed infectious TB (36). Decongestion should be considered as a structural component of TB prevention where overcrowding undermines clinical interventions.

Release should be managed as a high-risk clinical transition, not as an administrative endpoint. Post-release support should move beyond passive referral and include case management, peer navigation, transportation assistance, reminder systems, phone follow-up, and feedback from the receiving provider when appropriate. Evidence from TB infection, HIV, and other post-release care models supports active transition support over standard referral alone (81, 91, 94).

Prison TB programs should also be integrated with HIV and substance use services wherever these conditions are prevalent. For people living with HIV, this includes routine voluntary HIV testing, ART initiation or continuation, TB screening, TB preventive treatment when eligible after active TB has been excluded, LF-LAM use in appropriate clinical subgroups, and coordinated release planning for both TB and HIV care (91). For incarcerated persons with substance use disorders, TB treatment should be linked to addiction medicine, mental health care, harm reduction services consistent with national policy, and social support after release.

Peer educator programs can complement professional health services, particularly in resource-limited prisons where health staff are insufficient for continuous symptom recognition, health education, and referral (95). Peer programs should be implemented with safeguards: training, supervision, confidentiality protections, clear referral pathways, non-coercive participation, and appropriate recognition mechanisms. Peer educators should not be used as substitutes for professional diagnosis, infection prevention, or clinical treatment, but as part of a broader care and detection strategy (96, 97).

Prison TB control also requires reliable data. All people screened, diagnosed, treated, transferred, released, or lost to follow-up should be captured in a surveillance system that links prison health services with national TB programs. Prison status should be recorded as a relevant epidemiological and programmatic variable, while protecting confidentiality and avoiding stigmatization. Data systems should allow programs to monitor cascade indicators, including time from screening to diagnosis, time to treatment initiation, treatment completion, transfer outcomes, post-release linkage, and drug-resistance patterns (7, 91).

The overarching principle is that prison TB control should be evaluated not by isolated activity counts, such as the number of screening events performed, but by completion of the full prevention and care cascade: reduced exposure, earlier diagnosis, appropriate treatment, continuity after release, and integration with national TB surveillance.

## 10 Research and policy priorities

The next generation of prison TB research should move beyond detection yield alone and evaluate complete prevention and care cascades. Research should prioritize outcomes that matter for both individual care and public health impact: time to diagnosis, treatment initiation, treatment completion, continuity after transfer or release, recurrence, mortality, drug resistance, transmission, and integration with national TB surveillance.

Five research priorities are particularly urgent. First, rigorous evaluations of structured post-release case management for people receiving active TB treatment are needed. Pragmatic randomized trials, stepped-wedge designs, quasi-experimental evaluations, or registry-based linkage studies should assess treatment completion, treatment interruption, time to linkage with community TB services, recurrence, mortality, and, where feasible, post-release TB incidence or transmission (21). Second, prison-specific evaluations of shorter MDR/RR-TB regimens are needed, including BPaLM and related all-oral regimens for eligible patients. These studies should assess eligibility, resistance patterns, fluoroquinolone resistance, adverse-event monitoring, drug–drug interactions, treatment completion, acquired resistance, recurrence, and continuity after transfer or release (98, 99).

Third, implementation science studies should examine why interventions that appear effective under supported conditions often fail to achieve similar results at scale (100). Priority topics include fidelity of CXR/CAD screening, Xpert access and result return, medication supply, staffing models, confidentiality, peer educator supervision, transfer documentation, and prison–community data linkage (91). Fourth, diagnostic accuracy and implementation studies of LF-LAM are needed among incarcerated people living with HIV, particularly those with advanced immunosuppression, severe illness, or inability to produce sputum (101). Fifth, adequately powered studies of TB infection screening and treatment in incarcerated populations are needed, including completion of shorter preventive regimens, adverse events, post-release continuation, cost-effectiveness, and subsequent active TB incidence where feasible (92).

National TB strategic plans should explicitly include prisons and other detention settings as priority environments for TB prevention and care. This designation should be accompanied by dedicated funding, named program responsibility, measurable indicators, and routine reporting to national TB surveillance systems. Prison TB programs should be integrated with national TB programs rather than managed as isolated correctional health activities. Minimum policy requirements should include standardized entry and periodic screening strategies adapted to local burden, access to rapid molecular diagnosis and drug-resistance testing, uninterrupted treatment supply, infection prevention standards, TB/HIV integration, and formal transfer and release protocols (7).

International technical agencies should consider developing consolidated operational guidance for prison TB control that reflects contemporary diagnostic and treatment options, including Xpert MTB/RIF or Ultra, CXR/CAD, LF-LAM for selected people living with HIV, shorter TB infection regimens, and shorter all-oral MDR/RR-TB regimens. Such guidance should cover the full care pathway: screening, diagnosis, treatment, prevention, environmental control, data systems, transfer, release, and community linkage. Financing partners should support prison health-system strengthening as part of TB, HIV, and health security investments, including data systems, staff training, case management, ventilation improvements, peer programs, occupational health measures, and continuity-of-care mechanisms (10).

Finally, ministries of health and justice should jointly address overcrowding as a determinant of airborne TB transmission. Decongestion strategies—such as reducing unnecessary pre-trial detention, expanding appropriate non-custodial alternatives, improving case-processing efficiency, and ensuring medically appropriate release planning—should be evaluated as structural components of TB prevention, particularly in facilities where overcrowding undermines the effectiveness of clinical interventions. The central policy goal should be accountability across the full prison–community TB cascade: reduced exposure, timely diagnosis, appropriate treatment, completion or confirmed linkage after release, and integration of prison TB outcomes into national surveillance (21, 102).

## 11 Limitations of this review

This review has limitations. It was designed as a structured narrative review rather than a systematic review or scoping review, and therefore the protocol was not registered in PROSPERO. We did not perform duplicate independent screening, formal risk-of-bias assessment, quantitative synthesis, GRADE certainty assessment, or produce a PRISMA flow diagram. Although the review was informed by systematic-review principles and a structured evidence-mapping approach, the search was not designed to be exhaustive in the manner of a systematic review, no fully reproducible search log was generated, and selection of evidence involved expert judgment. Therefore, selection bias cannot be fully excluded, and some relevant publications may not have been captured.

The prison TB literature is heterogeneous across geography, epidemiological burden, prison type, diagnostic capacity, and health-system organization, which limits generalizability. Several important areas—including LF-LAM implementation, MDR/RR-TB treatment in correctional settings, post-release continuity for active TB, occupational TB risk among prison staff, and women’s prisons or immigration detention centers—remain supported by limited direct evidence. Some conclusions rely on mathematical modeling, policy guidance, or indirect evidence from adjacent fields such as HIV care, TB infection treatment, and substance use treatment continuity. These forms of evidence are useful for informing mechanisms and program design but should not be interpreted as direct estimates of prison-specific intervention effectiveness. Finally, because prison health systems are shaped by legal, political, and resource contexts, operational recommendations must be adapted locally.

## 12 Conclusion

Tuberculosis in prisons remains a preventable, diagnosable, and treatable disease that continues to cause avoidable morbidity, mortality, and transmission within and beyond correctional facilities. Its persistence reflects not only gaps in biomedical tools, but also the fragmentation of diagnostic, therapeutic, environmental, surveillance, and continuity-of-care systems. In many settings, prison health services remain under-resourced, poorly integrated with national TB programs, and insufficiently connected to community care after release.

The evidence reviewed in this article shows that effective tools are available. Digital chest radiography with computer-aided detection can improve case detection in high-burden settings when linked to confirmatory testing. Molecular diagnostics can rapidly confirm TB and detect rifampicin resistance. Shorter regimens for TB infection can improve completion compared with longer isoniazid-based regimens. Ventilation, safer spaces, and reduced occupancy can lower exposure intensity. Peer educator models can improve case detection in resource-limited settings. Structured post-release support is biologically and programmatically justified, although direct intervention evidence for active TB remains limited.

These tools are insufficient when implemented as disconnected activities. Screening must lead to diagnostic confirmation, treatment initiation, infection prevention action, and outcome reporting. Treatment support inside prison must be linked to transfer and release planning. TB, HIV, and substance use services should be integrated where these conditions overlap. Ventilation improvements are unlikely to achieve their full effect if extreme overcrowding persists. Surveillance systems must capture incarceration status, transfers, treatment outcomes, drug resistance, and post-release linkage.

The central conclusion of this review is that sustainable prison TB control requires moving from isolated interventions to an integrated prevention and care cascade. This cascade must link structural risk reduction, active case finding, rapid diagnosis, effective treatment, TB/HIV integration, environmental controls, data systems, and continuity after release. The evidence base is sufficient to justify immediate strengthening of prison TB control, while also making clear that further research is needed on post-release continuity, MDR/RR-TB treatment implementation, diagnostic cascade completion, LF-LAM use among incarcerated people living with HIV, and scalable implementation models.

Ultimately, prison TB control should be judged not by whether screening campaigns are conducted or diagnostic tools are procured, but by whether people are protected from avoidable exposure, diagnosed promptly, treated effectively, linked to care after release, and counted within national TB surveillance and accountability systems.
